# The effect of granulocyte colony‐stimulating factor dose and administration interval after allogeneic hematopoietic cell transplantation on early engraftment of neutrophil and platelet

**DOI:** 10.1002/jcla.24060

**Published:** 2021-10-21

**Authors:** Leila Noorazar, Hossein Bonakchi, Ghazaleh Sankanian, Sayeh Parkhideh, Maryam Salimi, Abbas Hajifathali, Reza Mirfakhraie, Elham Roshandel

**Affiliations:** ^1^ Hematopoietic Stem Cell Research Center Shahid Beheshti University of Medical Sciences Tehran Iran

**Keywords:** allo‐HSCT, early engraftment, neutrophil, platelet, post‐G‐CSF

## Abstract

**Background:**

Hematopoietic stem cell transplantation (HSCT) is one of the treatments for hematologic malignancies. Numerous factors affect the HSCT outcome. The purpose of this study was to investigate the effect of post‐HSCT administration of granulocyte colony‐stimulating factor (post‐G‐CSF) on early neutrophil and platelet engraftment in allogeneic HSCT (allo‐HSCT).

**Material & methods:**

The study was performed on 76 patients diagnosed with AML and ALL. All patients underwent allo‐HSCT at Taleghani stem cell transplantation center, Tehran, Iran, from February 2016 to December 2018. Chemotherapy regimens based on patients' conditions were selected between myeloablative and reduced‐intensity regimens.

**Results:**

Statistical analysis revealed that the number of administered G‐CSF units after HSCT was a time‐dependent variable. Statistical analysis before day +11 reported that patients who received G‐CSF <14 units had three times better early neutrophil engraftment than those with G‐CSF ≥14 (CI 95%, AHR = 3.03, *p*:0.002). CD3+ cells count <318.5 × 10^6^/kg was associated with fast platelet engraftment (CI 95%, AHR 2.28, *p*:0.01).

**Conclusion:**

In this study, post‐G‐CSF stimulation was associated with early engraftment in a time‐ and dose‐dependent manner. Administration of G‐CSF beyond 14 units resulted in adverse effects on neutrophil early engraftment. It also appeared that with a reduction in CD3+ cell counts, the likelihood of GVHD decreases, and platelet engraftment occurs earlier. Further investigations in the future are required to determine the factors affecting the process of early engraftment.

## Graphical abstract

After allogenic hematopoietic stem cell transplantation (HSCT), granulocyte colony stimulating factor (GCSF) is administrated to improve the neutrophil and platelet recovery. If the number of injected GCSF is <14 units until +11 day, the rate of early neutrophil engraftment will be three times better than the injected GCSF is ≥14 units.

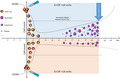

## 1 INTRODUCTION

Allogeneic hematopoietic stem cell transplantation (allo‐HSCT) is a promising treatment in patients with blood disorders.^1^ It has been found that successful engraftment is critical in decreasing the relapse risk. Hematopoietic stem cells (HSCs) privilege over residual leukemic cells in the competition for limited spaces of bone marrow niche reduces the risk of disease recurrence.^2^, ^3^ Successful hematopoietic recovery can also decrease the hospitalization period and reduce transfusion requirements.^4^ Efficient engraftment occurs through three steps: i) migration of infused HSCs toward bone marrow microvessels through adhesion molecules and their expression on the surface of endothelial cells; ii) stem cells homing to the bone marrow niche via interactions with homing receptors; iii) proliferation and differentiation of HSCs to various blood cells.^5^ Many factors may influence the kinetics of engraftment after allo‐HSCT, including the number of HSCs and T cells, subset of T cells, infections, graft‐versus‐host disease (GVHD) prophylaxis, post‐transplant granulocyte colony‐stimulating factor (G‐CSF) administration, and the disease status.^4^, ^6^, ^7^, ^8^ Cancer therapy‐related neutropenia is the most common complication resulting from chemotherapy toxicity.^9^ Before the HSCT, patients receive high‐dose chemotherapy regimens, which have myelosuppressive effects. Febrile neutropenia or post‐HSCT neutropenia makes patients susceptible to life‐threatening opportunistic infections.^10^, ^11^ Administration of G‐CSF after HSCT stimulates granulopoiesis and augments neutrophil recovery leading to a decrease in post‐HSCT infections. G‐CSF can stimulate the proliferation and differentiation of granulocyte precursors and release mature neutrophils to blood circulation, preventing infectious complications following neutropenic states.^12^ G‐CSF induces hematopoietic stem and progenitor cells (HSPCs) mobilization through different mechanisms. By receiving the G‐CSF signal, bone marrow monocytes suppress the osteoblast cells and disrupt the CXCL12‐CXCR4 axis, so HSCs detach from osteoblasts in the endosteal niche and release to peripheral blood. Other factors that involve in HSC mobilization/homing include c‐kit/kit‐ligand, vascular cell adhesion molecule (VCAM)‐1/very late antigen (VLA‐4), urokinase plasminogen activator (uPA)/uPA‐receptor, neutrophil‐derived proteases, and complement system components.^13^, ^14^ Neutrophil engraftment is defined as the first day of three consecutive days in which the absolute neutrophil count (ANC) is ≥0.5 × 10^9^/L, whereas platelet engraftment is defined as at least seven consecutive days without platelet transfusion in which the platelet count is >20 × 10^9^/L.^15^, ^16^, ^17^

To the best of our knowledge, previous literature in this field shows discrepancies about the best time to initiate G‐CSF administration after HSCT. Besides, there is no solid evidence suggesting the optimum number of G‐CSF units that can accelerate and improve neutrophil and platelet engraftment. Therefore, in this study, we evaluated the effect of post‐ HSCT G‐CSF administration on neutrophil and platelet early engraftment in allo‐HSCT patients.

## 2 METHODS

### 2.1 Patients

Seventy‐six patients who underwent allo‐HSCT between February 2016 and December 2018 at Taleghani hospital were enrolled in this retrospective study. The study was approved by the ethical committee of Shahid Beheshti University of Medical Sciences, and all patients signed the informed consent. The demographic characteristics of the patients are displayed in Table 1. The inclusion criteria for this study is to reach complete remission (CR) or partial remission (PR) status before starting the transplant procedure. The patient's diagnosis was divided into acute lymphoblastic leukemia (ALL) and acute myeloid leukemia (AML). Before the admission, the status of all patients was checked by cardiology, pulmonology, otolaryngology, psychology, and dentistry specialists. Besides, a broad panel of viral infections including toxoplasma, Epstein‐Barr virus, varicella‐zoster virus, hepatitis‐B and ‐C, and cytomegalovirus (CMV) was evaluated. None of the patients had an active infection. The patients' medical records were reviewed to collect data about age, gender, date of diagnosis, blood group, results of human leukocyte antigen (HLA) typing, previous treatment, and disease status. All patients received peripheral blood stem cells from an HLA full‐matched (8/8) sibling donor.

**TABLE 1:** Clinical characteristics of patients (N = 76)

Characteristics	Mean ± SD/Median (Range)/Frequency (%)	Missing
Recipient age, year	34.33 ± 11.23	3 (3.9)
Donor age, year	32.14 ± 11.50	5 (6.6)
Gender, M:F		
Recipient	41 (53.9):35 (46.1)	0 (0)
Donor	48 (63.2):27 (35.5)	1 (1.3)
Gender pairing match		
Match	36 (47.4)	1 (1.3)
Mismatch	39 (51.3)	
Diagnosed disease		
AML	53 (69.7)	3 (3.9)
ALL	20 (26.3)	
Recipient CMV activation Neutropenia phase IgG		
Positive	4 (5.3)	0 (0)
Recipient CMV activation 30 days after HSCT phase IgG		
Positive	9 (11.8)	0 (0)
ABO Match		
Match	37 (48.7)	16 (21.1)
Mismatch	23 (30.3)	
Recipient BMI		
<18.5^a^	0 (0)	
18.5–25	26 (34.2)	18 (23.7)
25–30	25 (32.9)	
>30	7 (9.2)	
Donor BMI		
<18.5	3 (3.9)	
18.5–25	16 (21.1)	35 (46.1)
25–30	15 (19.7)	
>30	7 (9.2)	
Infused cell dose/kg		
CD3	318.5 (3.80–700)	10 (13.2)
CD34	4 (1.7–12.3)	10 (13.2)
MNC	5.75 (2.9–11.9)	9 (11.8)
Cell types in apheresis product %		
CD3	370 (110–1000)	19 (25)
CD34	4.35 (1–12.5)	10 (13.2)
MNC	6.25 (0.9–25.2)	2 (2.6)
Number of injected GCSF after HSCT	14 (3–60)	2 (2.6)
Schedule of GCSF after HSCT		
Daily	44 (57.9)	5 (6.6)
Bi‐Daily	27 (35.5)	
Chemotherapy type		
MAC1	15 (19.7)	
MAC2	45 (59.2)	2 (2.6)
MAC3	5 (6.6)	
RIC	9 (11.8)	
Diagnosis‐HSCT interval, Year		
≤1	39 (51.3)	15 (19.7)
> 1	22 (28.9)	
Mean glucose value		
<100	2 (2.3)	
100–124	13 (17.1)	1 (1.3)
≥125	60 (78.9)	
Blood Group A:B:AB:O		
Recipient	16 (21.1):14 (18.4):6 (7.9):24 (31.6)	16 (21.1)
Donor	15 (19.7):12 (15.8):11 (14.5):19 (25)	19 (25)
Time of engraftment, Failure		
Neutrophil ≥0.5 × 10^3^/μl	10 (8–40)	5 (6.6)
Platelet ≥20 × 10^3^/μl	11 (5–42)	16 (21.1)
Baseline‐blood count donor characteristics		
WBC count(×10^9^/L)	13500 (4400–51800)	13 (17.1)
Hb	14.6 (4.35–47.7)	15 (19.7)
HCT (%)	41.18 ± 4.47	38 (50)
PLT (×10^9^/L)	252419.35 ± 66615.49	14 (18.4)
Pre‐PBSC donor characteristics		
WBC count(×10^9^/L)	23235.63 ± 7794.65	13 (17.1)
Hb	14.82 (8.68–47.7)	15 (19.7)
HCT (%)	41.92 (34.1–51.20)	38 (50)
PLT (×10^9^/L)	242666.12 ± 60777.57	14 (18.4)

Abbreviations: ALL, acute lymphoid leukemia; AML, acute myeloid leukemia; BMI, body mass index; CMV, cytomegalovirus; Hb, hemoglobin; HCT, hematocrit; HSCT, hematopoietic stem cell transplantation; MAC, myeloablative condition; MNC, mononuclear cell; PLT, platelet; RIC, reduced‐intensity conditioning; WBC, white blood count.

^a^ Omitted from analysis.

### 2.2 Chemotherapy regimens

Generally, our patients received conditioning regimens in four categories. Myeloablative conditioning (MAC)‐1 regimen consisted of busulfan and cyclophosphamide (Bu/Cy) as 0.8 mg/kg intravenous busulfan (Bu) every 6 h for 4 days and 60 mg/kg cyclophosphamide (Cy) for 2 days. In the MAC‐2 (Bu/Flu) regimen, fludarabine (Flu) 30 mg/m^2^ once a day for 5 days was prescribed instead of cyclophosphamide. In the MAC‐3 (Bu/Flu/ATG) regimen, 60 mg/kg of anti‐thymocyte globulin (ATG) was added to the MAC‐2 regimen, but the total dose of busulfan was reduced to 12 mg/kg. These regimens were used in patients under 45 years and patients without comorbidity. Reduced‐intensity conditioning (RIC) regimen, comprised of 30 mg/m^2^ fludarabine IV for 5 days and 100 mg/m^2^ lomustine (1‐[2‐chloroethyl]‐3‐cyclohexyl‐l‐nitrosourea [CCNU]) orally for 2 days, was used for the rest of the patients. GVHD prophylaxis was prescribed based on our center's protocol, as mentioned in our previous reports.^18^, ^19^

### 2.3 Serum glucose level

Both fasting and non‐fasting whole‐blood glucose for each patient was measured daily. The patients were categorized to three groups based on their mean glucose level: a) <100 mg/dl, b) 100–124 mg/dl, c) ≥125 mg/dl.

### 2.4 Mobilization, collection, and laboratory processing method of stem cell

Donors received 5–10 μg/kg/d of G‐CSF subcutaneously for 4–5 days. The released stem cells to the peripheral bloodstream were collected by apheresis Spectra Optia (Terumo BCT). The CD34+ (human PE‐conjugated anti‐CD34, EXBIO) and CD3+ (human FITC‐conjugated anti‐CD3, Beckman Coulter) cell counts were measured by flow cytometry (Attune NxT, Invitrogen). Cell viability was performed using trypan blue staining on a hematocytometer chamber. RBC depletion, plasma reduction, or both of them were performed in case of ABO mismatch. For RBC depletion, hydroxyl ethyl starch 6% [HES] (GRIFOLS) was used while the plasma reduction process was performed by washing with 0.9% saline solution three times.

### 2.5 Engraftment evaluation

Hematopoietic recovery was defined based on daily complete blood cell counts. Myeloid engraftment was considered to occur on the first day of three consecutive days in which absolute neutrophil count (ANC) was ≥0.5 × 10^9^/L (15). Platelet engraftment was defined as at least seven consecutive days without platelet transfusion in which the platelet count was >20 × 10^9^/L.^16^, ^17^

### 2.6 Statistical analysis

In the descriptive analysis, the categorical variables with the frequencies and percentages, the normally distributed continuous variables with mean ± SD, and the non‐parametric variables with median and range were reported. The endpoint of the study was early platelet and neutrophil engraftments. The log‐rank test was applied for the univariate analysis and comparing the engraftments probability among the groups. Risk factors with significance levels less than 0.2 in univariate analysis were considered in the multiple Cox proportional hazard model in which a backward method was employed for feature selection. The significance level in the multiple analysis was set at 0.05. The proportional hazards assumption was performed using the score process plot and the Kolmogorov‐type supremum test (the significance level was 0.05). The association of CD3+, 'CD34+ and mononuclear cell (MNC) in apheresis product with donor characteristics for normal and non‐parametric variables was examined by Pearson and Spearman correlation coefficients, respectively. The significance level was set at ≤0.05. The calculations were carried out using SAS (version 9.4; SAS Institute Inc).

## 3 RESULTS

### 3.1 Patients

A total of 76 patients were included in this study. The clinical characteristics of the patients are shown in Table 1. The medians of infused CD3+, CD34+, and mononuclear cells were 318.5 (range: 3.8–700) × 10^6^/kg, 4 (range: 1.7–12.3) × 10^6^/kg, and 5.75 (range: 2.9–11.9) × 10^6^/kg, respectively. The mean age of the patients was 34.33 ± 11.23. Fifty‐three (69.7%) patients had AML and 20 (26.3%) patients had ALL. The medians of neutrophil and platelet engraftment days were 10 and 11 days, in the order given.

### 3.2 Univariate analysis

The medians of infused CD34+, ‘CD3+, and MNC dosage were selected as the cut‐off point, and the patients were divided into two groups based on these thresholds for the analysis. In univariate analysis, the effects of risk factors such as gender, age, blood group, BMI of donors and patients, gender parity, CMV reactivation in neutropenia phase and within 30 days post‐transplant, ABO blood group matching status, diagnosis, number and timing of injected G‐CSF unit after HSCT (daily or bidaily), chemotherapy type, the interval between diagnosis to HSCT, glucose level, and disease status before HSCT on early neutrophil and platelet engraftments were examined. For early neutrophil engraftment, recipient and donor gender, recipient and donor age, CMV activation in neutropenia phase and 30 days after HSCT, ABO match, recipient and donor blood group, donor BMI, chemotherapy type, diagnosis‐HSCT interval, glucose value, disease status before HCT, CD3 × 10^6^/kg, CD34 × 10^6^/kg, and MNC × 10^8^/kg were not significant. Also, for early platelet engraftment, recipient and donor gender, recipient and donor age, gender pairing, CMV activation in neutropenia phase and 30 days after HSCT, ABO match, donor blood group, diagnosed disease, donor BMI, schedule of GCSF after HSCT, diagnosis‐HSCT interval, glucose value, disease status before HCT, and MNC × 10^8^/kg were not significant. There was a significant difference among CD3+ groups in early platelet engraftment (*p*‐value = 0.05). There was a significant difference among CD34+ groups in early platelet engraftment (*p*‐value = 0.03). Among other risk factors, early neutrophil engraftment in gender pairing groups (*p*‐value = 0.17), disease type groups (*p*‐value = 0.003), recipient's BMI (*p*‐value = 0.01), number of injected G‐CSF unit after HSCT groups (*p*‐value = 0.005), and protocol of G‐CSF administration after HSCT (*p*‐value = 0.15) was statistically significant (Table 2). Also, early platelet engraftment in recipient's blood groups (*p*‐value = 0.14), recipient's BMI (*p*‐value = 0.12), number of injected GCSF after HSCT groups (*p*‐value = 0.06), and chemotherapy type (*p*‐value = 0.03) were statistically significant (Table 3).

**TABLE 2:** The effect of risk factors on early neutrophil engraftment

			Univariate^a^		Multiple^b^	
Variables	Day≤12	Day>12	Early neutrophil engraftment (%)	*p*‐value	AHR (95% CI)	*p*‐value
Recipient gender (%)						
Male	30 (54.5)	3 (60)	88	0.37		
Female (RL)	25 (45.5)	2 (40)	89			
Donor gender (%)						
Male	36 (65.5)	3 (60)	87	0.71		
Female (RL)	19 (34.5)	2 (40)	90			
Recipient age (%)						
<34	26 (47.3)	4 (80)	81	0.43		
≥34 (RL)	29 (52.7)	1 (20)	94			
Donor age (%)						
<32	22 (42.3)	2 (40)	91	0.51		
≥32 (RL)	30 (57.7)	3 (60)	88			
Gender pairing (%)						
Match	29 (52.7)	1 (20)	93	0.17*		NS
Mismatch (RL)	26 (47.3)	4 (80)	83			
CMV activation of neutropenia phase (%)						
Positive	2 (3.6)	0 (0)	100	0.77		
Negative (RL)	53 (96.4)	5 (100)	87			
CMV activation of 30 days after HSCT (%)						
Positive	8 (14.5)	1 (20)	89	0.88		
Negative (RL)	47 (85.5)	4 (80)	88			
ABO Match (%)						
Match	27 (49.1)	2 (66.7)	90	0.73		
Mismatch (RL)	17 (30.9)	1 (33.3)	94			
Recipient Blood Group (%)						
A	11 (24.5)	0 (0)	100	0.22		
B	9 (20)	1 (33.3)	90			
AB	5 (13.3)	1 (33.3)	83			
O (RL)	19 (42.2)	1 (33.3)	90			
Donor Blood Group (%)						
A	11 (20)	0 (0)	100	0.72		
B	9 (16.4)	1 (50)	90			
AB	9 (16.4)	0 (0)	100			
O (RL)	13 (23.6)	1 (50)	85			
Diagnosed disease (%)						0.01**
AML	40 (72.7)	5 (100)	84	0.003*	0.46 (0.24–0.87)	0.01
ALL (RL)	15 (27.3)	0 (0)	100		‐	‐
Recipient BMI (%)						
18.5–25	21 (38.2)	0 (0)	100	0.01*		NS
25–30	17 (30.9)	3 (100)	80			
> 30 (RL)	5 (9.1)	0 (0)	100			
Donor BMI (%)						
<18.5	2 (3.6)	0 (0)	100			
18.5–25	12 (21.8)	1 (33.3)	92	0.85		
25–30	11 (20)	1 (33.3)	91			
>30 (RL)	6 (10.9)	1 (33.3)	85			
Number of injected GCSF after HSCT						
All days (%)						
<14	27 (50)	2 (40)	93	0.005*		
≥14 (RL)	27 (50)	3 (60)	83			
< 11 days (%)				0.02*		0.002**
<14	23 (65.7)	0 (0)	47		3.03 (1.47–6.26)	0.002
≥14 (RL)	12 (34.3)	0 (0)	8		‐	‐
>11 days (%)				0.68		0.68
<14	4 (19)	2 (28.6)	66		0.79 (0.26–2.40)	0.68
≥14 (RL)	17 (81)	5 (71.4)	77		‐	‐
Schedule of GCSF after HSCT (%)						
Daily	32 (62.7)	2 (40)	91	0.15*		NS
Bi‐Daily (RL)	19 (37.3)	3 (60)	81			
Chemotherapy type (%)						
MAC 1	9 (16.4)	3 (60)	75	0.30		
MAC 2	35 (63.6)	1 (20)	91			
MAC 3	4 (7.3)	0 (0)	100			
RIC (RL)	7 (12.7)	1 (20)	87			
Diagnosis‐HSCT interval, Year (%)						
≤ 1	31 (68.9)	2 (50)	93	0.71		
>1 (RL)	14 (31.1)	2 (50)	81			
Mean glucose value (%)						
<100	2 (3.7)	0 (0)	100	0.68		
100–124	8 (14.8)	2 (40)	80			
≥125 (RL)	44 (81.5)	3 (60)	91			
Disease status before HCT (%)						
CR	39 (100)	15 (100)	70	NE		
PR(RL)	0 (0)	0 (0)	0			
CD3 × 10^6^/kg (%)						
<318.5	25 (45.5)	1 (20)	0.92	0.68		
≥318.5 (RL)	30 (54.5)	4 (80)	0.85			
CD34 × 10^6^/kg (%)						
<4	30 (54.5)	1 (20)	0.93	0.85		
≥4 (RL)	25 (45.5)	4 (80)	0.82			
MNC × 10^8^/kg (%)						
<5.75	26 (47.3)	1 (20)	0.96	0.55		
≥5.75 (RL)	29 (52.7)	4 (80)	0.81			

Abbreviations: AHR, Adjusted hazard ratio; RL, Reference level.

^a^ Log‐rank test.

^b^ Backward selection.

* Significant at 0.20.

** Significant at 0.05.

**TABLE 3:** The effect of risk factors on early platelet engraftment

			Univariate^a^		Multiple^b^	
Variables	Day≤12	Day>12	Early platelet engraftment (%)	*p*‐value	HR (95% CI)	*p*‐value
Recipient gender (%)						
Male	29 (56.9)	8 (50)	78	0.77		
Female (RL)	22 (43.1)	8 (50)	70			
Donor gender (%)						
Male	32 (62.7)	11 (68.8)	74	0.75		
Female (RL)	19 (37.3)	5 (31.3)	75			
Recipient age (%)						
<34	25 (50)	7 (46.7)	75	0.91		
≥34 (RL)	25 (50)	8 (53.3)	75			
Donor age (%)						
<32	20 (39.2)	6 (40)	76	0.83		
≥32 (RL)	29 (56.9)	9 (60)	73			
Gender pairing (%)						
Match	24 (47.1)	9 (56.3)	69	0.42		
Mismatch (RL)	27 (52.9)	7 (43.8)	79			
CMV activation of neutropenia phase (%)						
Positive	3 (5.9)	1 (6.3)	75	0.84		
Negative (RL)	48 (94.1)	15 (93.8)	74			
CMV activation of 30 days after HSCT (%)						
Positive	6 (11.8)	2 (12.5)	75	0.96		
Negative (RL)	45 (88.2)	14 (87.5)	74			
ABO Match (%)						
Match	27 (64.3)	8 (66.7)	74	0.93		
Mismatch (RL)	15 (35.7)	4 (33.3)	78			
Recipient Blood Group (%)						
A	14 (31.8)	1 (8.3)	93	0.14*		NS
B	8 (18.2)	4 (33.3)	63			
AB	5 (11.4)	2 (16.7)	50			
O (RL)	17 (38.6)	5 (41.7)	77			
Donor Blood Group (%)						
A	9 (22)	3 (30)	75	0.44		
B	8 (19.5)	4 (40)	66			
AB	8 (19.5)	1 (10)	77			
O (RL)	16 (39)	2 (20)	88			
Diagnosed disease (%)						
AML	36 (72)	10 (66.7)	76	0.65		
ALL (RL)	14 (28)	5 (33.3)	73			
Recipient BMI (%)						
18.5–25	18 (43.9)	6 (50)	75	0.12*		NS
25–30	16 (39)	6 (50)	68			
>30 (RL)	7 (17.1)	0 (0)	100			
Donor BMI (%)						
<18.5	1 (3.6)	1 (12.5)	50	0.85		
18.5–25	10 (35.7)	2 (25)	83			
25–30	11 (39.3)	4 (50)	66			
>30 (RL)	6 (21.4)	1 (12.5)	85			
Number of injected GCSF after HSCT (%)						
<14	29 (56.9)	6 (40)	82	0.06*		NS
≥14 (RL)	22 (43.1)	9 (60)	67			
Schedule of GCSF after HSCT (%)						
Daily	34 (69.4)	8 (57.1)	80	0.30		
Bi‐Daily(RL)	15 (30.6)	6 (42.9)	66			
Chemotherapy type (%)						
MAC 1	5 (9.8)	6 (40)	45	0.03*		NS
MAC 2	37 (72.5)	6 (40)	86			
MAC 3	4 (7.8)	0 (0)	75			
RIC (RL)	5 (9.8)	3 (20)	62			
Diagnosis‐HSCT interval, Year (%)						
≤1	30 (69.8)	7 (53.8)	78	0.21		
>1 (RL)	13 (30.2)	6 (46.2)	68			
Mean glucose value (%)						
<100	2 (3.9)	0 (0)	100	0.34		
100–124	7 (13.7)	6 (37.5)	53			
≥125 (RL)	42 (82.4)	10 (62.5)	71			
Disease status before HCT (%)						
CR	43 (100)	11 (100)	77	NE		
PR (RL)	0 (0)	0 (0)	0			
CD3 × 10^6^/kg (%)				0.05*		0.01**
<318.5	27 (52.9)	4 (25)	0.87		2.28(1.17–4.42)	0.01
≥318.5 (RL)	24 (47.1)	12 (75)	0.63		‐	‐
CD34 × 10^6^/kg (%)						
<4	31 (60.8)	4 (25)	0.85	0.03*		NS
≥4 (RL)	20 (39.2)	12 (75)	0.62			
MNC × 10^8^/kg (%)						
<5.75	24 (47.1)	7 (43.8)	0.77	0.63		
≥5.75 (RL)	27 (52.9)	9 (56.3)	0.72			

Abbreviations: CR, complete remission; PR, partial remission; RL, Reference Level.

^a^ Log‐rank test.

^b^ Backward selection.

* Significant at 0.20.

** Significant at 0.05.

### 3.3 Multiple analysis

For early neutrophil engraftment with backward selection method, the diagnosed disease and the number of G‐CSF injected after transplantation were significantly effective. The score process plot and the Kolmogorov‐type supremum test suggest that the proportional hazard assumption was not satisfied for the number of injected G‐CSF units after transplantation variable, and it was time‐dependent. The survival curve based on the Cox model and contrast test showed that the hazard ratios were not constant before and after 11 days (data not shown). Therefore, the extended Cox model with two Heaviside functions was used. The results showed that AML patients had 54% delayed neutrophil engraftment compared to ALL patients. Also, the hazard ratio for the effect of the number of injected G‐CSF units after transplantation was significant [*p*‐value = 0.002] when the time was less than +11 days. The patients with less than 14 units of G‐CSF had three times better early neutrophil engraftment compared to patients with more than 14 G‐CSF units. The hazard ratio for the effect of the number of G‐CSF injected after transplantation was insignificant [*p*‐value = 0.68] when the time was more than +11 days (Table 2). For early platelet engraftment with the backward selection method, the infused CD3+ dosage was effective. The patients who received <318.5 × 10^6^ CD3+ cells/kg were more likely to have a successful early platelet engraftment, compared to those who received ≥318.5 × 10^6^ CD3+ cells/kg [*p*‐value = 0.01] (Table 3).

### 3.4 Association of CBC parameters of donors with the infused CD3, CD34, and MNC dosage

**FIGURE 1:**
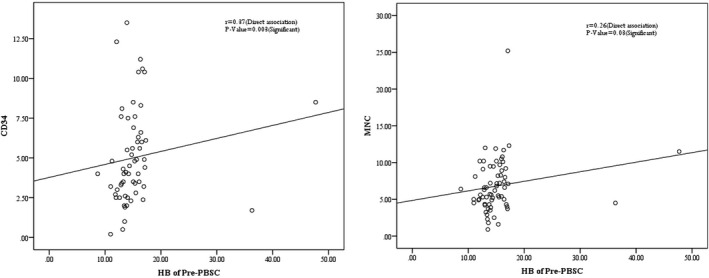
Association pre‐PBSC hemoglobin with CD34 and MNC

As shown in Table 4, the association between donor hemoglobin level on the day of peripheral blood stem cell harvesting and MNC count in apheresis product (known as baseline‐PBSC) was nearly significant *p*‐value = 0.057). There is a significant direct relationship between the donor hemoglobin level in pre‐PBSC and CD34+ cell count in apheresis product (*p*‐value = 0.01). Also, there is a significant direct correlation between the donor hemoglobin level in pre‐PBSC and MNC count in apheresis product (*p*‐value = 0.006). The CD34+ cell count and MNC count were increased by increasing donor hemoglobin level in pre‐PBSC (Figure 1).

**TABLE 4:** Association characteristics of donors with CD3, CD34, and MNC in apheresis product

	CD3 × 10^6^/kg	CD34 × 10^6^/kg	MNC × 10^8^/kg
Donor age	0.24^r^	0.11^r^	0.07^r^
	0.07^P^	0.37^P^	0.53^P^
Baseline‐blood count			
WBC count(×10^9^/L)	0.24^r^	0.11^r^	0.12^r^
	0.12^P^	0.44^P^	0.36^P^
Hb	0.08^r^	0.19^r^	0.22^r^
	0.56^P^	0.13^P^	0.057^P^
HCT (%)	0.009^r^	0.16^r^	0.19^r^
	0.95^P^	0.26^P^	0.14^P^
PLT (×10^9^/L)	−0.03^r^	−0.15^r^	−0.11^r^
	0.81^P^	0.22^P^	0.36^P^
Pre‐PBSC			
WBC count(×10^9^/L)	0.18^r^	0.04^r^	0.21^r^
	0.76^P^	0.26^P^	0.10^P^
Hb	0.24^r^	0.33^r^	0.35^r^
	0.09^P^	0.01* ^P^	0.006* ^P^
HCT (%)	−0.17^r^	0.28^r^	−0.01^r^
	0.36^P^	0.13^P^	0.92^P^
PLT (×10^9^/L)	−0.08^r^	−0.26^r^	−0.13^r^
	0.58^P^	0.054^P^	0.30^P^

Note

Underline, Borderline significant.

Abbreviation: Pre‐PBSC, preliminary peripheral blood cell count before stem cell collection. r:correlation coefficient, it shows how one variable affects another; p: P value, this item indicates the significance of the data relationship.

* Significant at 0.05.

## 4 DISCUSSION

Allo‐HSCT has been confirmed as a treatment for many hematologic disorders.^20^ Stem cell engraftment depends on various factors such as MNC and CD34+ cell count, HLA‐matching, ABO compatibility, age, sex, and BMI of donor and patient, disease status before HSCT, CMV reactivation, conditioning regimens, GVHD prophylaxis regimens, and various unknown factors. In this study, we aimed to determine the effects of these factors especially post‐G‐CSF administration on neutrophil and platelet early engraftment.

Pre‐transplant G‐CSF is used as a part of regimens to increase stem cells in peripheral blood for harvesting. The most common problem that affects patients after HSCT is infection due to prolonging the neutropenic phase.^21^ G‐CSF administration after HSCT could reduce the neutropenic phase duration, decrease the risk of life‐threatening infections, and accelerate the granulocyte recovery.^22^ We prescribed G‐CSF as intravenous (IV) infusion with a dose of 5–10 µg/kg/day on day one after HSCT until the ANC became ≥0.5 × 10^9^/L for three consecutive days. Our results showed that the median day of neutrophil and platelet engraftment was 10 and 11 days, respectively. Several reports demonstrated that G‐CSF administration after HSCT caused faster neutrophil engraftment by 1–6 days compared with the placebo group. Bishop et al. evaluated 54 patients with hematologic malignancies who were transplanted from sibling donors. They indicated that the median time to achieve neutrophil engraftment in patients who received 10 µg/kg filgrastim (recombinant human G‐CSF) daily was 11 days versus 15 days in the placebo group. The platelet engraftment also occurred 2 days earlier in the G‐CSF group relative to that of the placebo group (13 days and 15.5 days).^23^ Another report showed that starting 5 µg/kg G‐CSF administration on day +1 could reduce neutrophil engraftment time from 16 to 10.5 days in the G‐CSF group compared to the control group. Furthermore, the median time of hospitalization (18 vs. 24 days) and days on broad‐spectrum antibiotics (11 vs. 15 days) were significantly reduced with the administration of G‐CSF after HSCT.^24^ In a similar study, lenograstim (another recombinant human G‐CSF) decreased the length of myeloid recovery from 12.5 to 9 days after HSCT.^25^ However, documents were controversial about the time of platelet engraftment. We reported that platelet engraftment occurred 1 day later than neutrophils. In Bishop's study, filgrastim, used as a granulocyte growth factor, ameliorated the platelet engraftment for 2.5 days (13 vs. 15.5 days).^23^ Linch et al.^25^ showed that the use of lenograstim did not have any effects on the time of platelet engraftment, while Ringden et al.^26^ reported that patients who were treated with G‐CSF had slower platelet engraftment (>50 × 10^9^/L; 18 vs. 15 days). On the other hand, Özcan et al.^27^ declared that the rate of febrile events in the G‐CSF group was significantly lower than the control group (75% vs 100%).

The median number of post‐HSCT G‐CSF administered in our study was 14 units. According to multiple statistical analyses, G‐CSF exhibited a time‐dependent effect on neutrophil engraftment so that patients who received doses less than 14 units G‐CSF during the first 11 days post‐HSCT were more likely to have early neutrophil engraftment.

SDF‐1 (CXCL12) is an important member of the chemokine family, which plays a vital role in stem cell homing.^28^ In the HSCT process, conditioning regimen and chemotherapy before transplantation increase the secretion of SDF‐1 from osteoblast in the endosteal niche. The reciprocal link between SDF‐1 and CXCR4 begins HSC homing and engraftment after transplantation.^28^, ^29^ Our study revealed that injection of more than 14 G‐CSF units in 11 days after HSCT could be associated with a reduced occurrence of early neutrophil engraftment. Thompson et al.^30^ found that in patients undergoing auto‐HSCT, starting G‐CSF on the same day of HSCT caused faster recovery of hematological parameters. In addition, in patients undergoing HSCT, entering severe pancytopenia phase before transplantation increases opportunistic infection risk.^31^ Hence, it is recommended that G‐CSF should be used on the first day of cell transplantation. Accordingly, we started the G‐CSF at day +1 to prevent the possibility of infection. However, in other study, it was noted that there was no significant difference between the time of starting G‐CSF at day 0, +5, +10 and the time of neutrophil engraftment.^32^, ^33^ Therefore, with the mechanisms mentioned above, G‐CSF administration in the first days after transplantation can reduce the risk of opportunistic infections by affecting the proliferation and differentiation of HSCs to increase mature neutrophils. Nonetheless, according to our results, it seems that using more than 14 units of G‐CSF during 11 days after HSCT can impair the HSCs homing by preventing them from binding to the endosteal niche, thereby results in delaying the neutrophil engraftment.

Moreover, our study indicated that AML patients had a lower risk of early neutrophil engraftment than ALL group. To the best of our knowledge, patients with advanced stages of the disease are more likely to receive multi‐stage chemotherapy regimens, so they may have minimal residual leukemic cells before the time of HSCT that is called partial remission (PR). These patients will probably relapse after a while, and these conditions are more common in AML patients with a poorer prognosis than ALL patients.^34^ It was mentioned that several cycles of chemotherapy and also the leukemic cells that survived after chemotherapy caused the delay in HSC engraftment in AML patients.^35^, ^36^

Interestingly, the time of platelet engraftment in our study was independent of G‐CSF doses before day +11. However, Shimoda et al.^37^ reported that G‐CSF injection triggered platelet aggregation and led to transient thrombocytopenia in healthy donors before the apheresis process.

We could not conclude that CD34+ cell count had an association with the early neutrophil and platelet engraftment. Regardless, a high dose of CD34+ was nearly associated with rapid engraftment of neutrophils and platelets.^38^, ^39^ It has been reported that the CD34+ cell dose between 2 and 4 × 10^6^/kg of recipient weight was necessary for rapid engraftment.^39^ previous reports showed the critical role of CD3+ T cells in sustaining the engraftments.^40^, ^41^ The role of CD3+ cells in the occurrence of GVHD has already been proven.^42^ High numbers of CD3+ cells, HSCs, and monocytes in the graft limit the rate of graft rejection.^43^ In a study with 256 patients, Urbano‐Ispizua et al.^41^ concluded that the patients with less than 0.2 × 10^6^/kg CD3+ cells in the graft were at risk of graft failure. Rauofi et al.^44^ stated that the rate of cGVHD was decreased in patients with CD3+ <365 × 10^6^/kg compared to patients with CD3+ >365 × 10^6^/kg. Contrarily, Chang et al.^45^ declared that higher counts of CD3+ cells (median, 1.64 × 10^8^/kg) might be a guarantee to sustain the engraftment. Likewise, we demonstrated that patients who received <318.5 × 10^6^/kg CD3+ cells had early platelet engraftment, probably due to the low incidence of GVHD.

Our results showed that the amount of donor hemoglobin before stem cell isolation was significantly related to the count of CD34+ cells and MNCs in the graft product. Since the apheresis device is adjusted based on the physiological conditions of the donor before the apheresis procedure, including age, blood pressure, hemoglobin level, etc., it is possible that in donor with higher hemoglobin, the volume of blood entering the device set higher and therefore, there might be more amount of isolated CD34+ cells and MNCs per circulation.

The findings of this study showed that administration of G‐CSF in the first days after HSCT could accelerate the time of neutrophil engraftment. In fact, we suppose that higher doses of G‐CSF before the 11 days post‐HSCT result in faster hematologic reconstitution.

## AUTHOR CONTRIBUTIONS

A.H., R.M., E.R: conception and design; L.N., H.B.: acquisition of data, or analysis and interpretation of data S.K., L.N., H.B., M.S., S.P.: drafting the article or revising it critically for important intellectual content. All authors revised the final approval of the version to be submitted for publication.

## Data Availability

The data that support the findings of this study are available from the corresponding author upon reasonable request.
